# International expert consensus statement: surgical failure in obstructive sleep apnea

**DOI:** 10.1007/s11325-024-03162-6

**Published:** 2024-09-23

**Authors:** Giannicola Iannella, Annalisa Pace, Giuseppe Magliulo, Claudio Vicini, Rodolfo Lugo, Olivier M. Vanderveken, Nico de Vries, Kenny Pang, Eric Thuler, Ofer Jacobowitz, Michel Burihan Cahali, Joachim T. Maurer, Manuele Casale, Antonio Moffa, Fabrizio Salamanca, Federico Leone, Ewa Olszewska, Carlos O’connor Reina, Edilson Zancanella, Paul T. Hoff, Peter Baptista, Ahmed Yassin Bahgat, Madeline J. L. Ravesloot, Peter van Maanen, Andrew Goldberg, Marina Carrasco, Vikas K. Agrawal, Jerome R. Lechien, Andrea De Vito, Giovanni Cammaroto, Armando De Virgilio, Antonio Greco, Patrizia Mancini, Tiziano Perrone, Steve Amado, Uri Alkan, Ryan Chin Taw Cheong, Aurelio D’Ecclesia, Dorina Galantai, Anand RajuAnand, Christian Calvo-Henriquez, Salvatore Cocuzza, Michele Arigliani, Alberto Maria Saibene, Rosario Marchese Aragona, Antonino Maniaci

**Affiliations:** 1 Department of ‘Organi Di Senso’, University “Sapienza”, Viale Università 33, 00185 Rome, Italy; 2 Department ENT & Audiology, University of Ferrara, Ferrara, Italy; 3 Department of Otorhinolaryngology, Grupo Medico San Pedro, 64660 Monterrey, Mexico; 4 Faculty of Medicine and Health Sciences, Translational Neurosciences, University of Antwerp, Antwerp, Belgium; 5 Department of ENT, Head and Neck Surgery, Antwerp University Hospital, Edegem, Belgium; 6 Multidisciplinary Sleep Disorders Centre, Antwerp University Hospital, Edegem, Belgium; 7 Department of Orofacial Pain and Dysfunction, Academic Center for Dentistry Amsterdam (ACTA), University of Amsterdam, Amsterdam, The Netherlands; 8 Asia Sleep Centre, Singapore, Singapore; 9 Division of Sleep Surgery, Department of Otorhinolaryngology, University of Pennsylvania Perelman School of Medicine, Philadelphia, PA USA; 10 Sleep Department, ENT and Allergy Associates, New York, NY USA; 11 Departamento de Otorrinolaringologia, Hospital das Clínicas da Faculdade de Medicina da Universidade de São Paulo (HC-FMUSP), São Paulo, SP Brazil; 12 Division of Sleep Medicine, Department of Otorhinolaryngology, University Hospital Mannheim, Mannheim, Germany; 13 Integrated Therapies in Otolaryngology, Fondazione Policlinico Universitario Campus Bio-Medico, 00128 Rome, Italy; 14 Otorhinolaryngology Unit, San Pio X Hospital, 20159 Milan, Italy; 15 Department of Otolaryngology, Sleep Apnea Surgery Center, Medical University of Bialystok, 15-276 Bialystok, Poland; 16 Otorhinolaryngology Department, Hospital Quironsalud Marbella, Marbella, Spain; 17 State University of Campinas, Campinas, São Paulo, Brazil; 18 Department of Otolaryngology-Head and Neck Surgery, University of Michigan, Ann Arbor, MI USA; 19 Otorhinolaryngology Department, Clínica Universitaria de Navarra, Pamplona, Spain; 20 Department of Otorhinolaryngology-Head & Neck Surgery, Alexandria University, Alexandria, 5424041 Egypt; 21 Department of Otorhinolaryngology-Head and Neck Surgery, OLVG, Amsterdam, The Netherlands; 22 Division of Rhinology and Sinus Surgery, Department of Otolaryngology, Head and Neck Surgery, University of California, 2233 Post Street, Room 309, San Francisco, CA 94115-1225 USA; 23 Department of Otorhinolaryngology, Doctor Peset University Hospital, Valencia, Spain; 24 Speciality ENT Hospital, Thakur Complex, Kandivali (E), Mumbai, Maharashtra 400101 India; 25 Division of Laryngology and Broncho-Esophagology, Department of Otolaryngology-Head Neck Surgery, EpiCURA Hospital, UMONS Research Institute for Health Sciences and Technology, University of Mons (UMons), Mons, Belgium; 26 Department of Otolaryngology-Head and Neck Surgery, Forli Hospital, Forli, Italy; 27 Otorhinolaryngology Unit, Civil Hospital of Alghero, Alghero, Italy; 28 Maple Respiratory, Universidad del Rosario, Bogotá, Colombia; 29 Department of Otolaryngology Head and Neck Surgery, Rabin Medical Center, Petah Tikva, Israel; 30 Royal National ENT and Eastman Dental Hospitals, University College London Hospitals NHS, London, UK; 31 IRCCS ‘Casa Sollievo Della Sofferenza’, San Giovanni Rotondo, Italy; 32 Department of Otorhinolaryngology, Head and Neck Surgery, Bajcsy-Zsilinszky Hospital, Budapest, Hungary; 33 Department of ENT Surgery, PSG Hospitals, Coimbatore, India; 34 Rhinology and Sleep Apnea Unit, Otolaryngology Department, Hospital Complex of Santiago de Compostela, Santiago de Compostela, Spain; 35 Deparment of Medical, Surgical Sciences and Advanced Technologies G.F. Ingrassia, University of Catania, 95123 Catania, Italy; 36 Otolaryngology Unit, Vito Fazzi Hospital, Lecce, Italy; 37 Otolaryngology Unit, Santi Paolo E Carlo Hospital, Department of Health Sciences, Università Degli Studi Di Milano, Milan, Italy; 38 Otolaryngology Clinic, Department of Neurosciences, University of Padova, Padua, Italy; 39 Department of Otolaryngology, Enna Kore University, Enna, Italy

**Keywords:** Obstructive sleep apnea, Delphi method, Expert consensus, Clinical guidelines, Sleep disorder management

## Abstract

**Purpose:**

Upper airway (UA) surgery is commonly employed in the treatment of patients with obstructive sleep apnea (OSA). The intricate pathophysiology of OSA, variability in sites and patterns of UA collapse, and the interaction between anatomical and non-anatomical factors in individual patients may contribute to possible surgical failures. This clinical consensus statement aims to identify areas of agreement among a development group comprising international experts in OSA surgery, regarding the appropriate definition, predictive factors in patients, and management of surgical failure in OSA treatment.

**Methods:**

A clinical consensus statement (CCS) was developed using the Delphi method by a panel of 35 contributors from various countries. A systematic literature review adhering to PRISMA guidelines was conducted. A survey consisting of 60 statements was then formulated and presented to the experts.

**Results:**

Following two rounds of the Delphi process, consensus or strong consensus was achieved on 36 items, while 24 items remained without consensus. Specifically, 5 out of 10 statements reached consensus regarding on the 'Definition of Surgical Success/Failure after OSA Surgery'. Regarding the 'Predictive Factors of Surgical Failure in OSA Surgery', consensus was reached on 10 out of 13 statements. In the context of the 'Diagnostic Workup in OSA Surgery', consensus was achieved on 9 out of 13 statements. Lastly, in 'Treatment in Surgical Failure Cases', consensus was reached on 12 out of 24 statements.

**Conclusion:**

The management of OSA after surgical failure presents a significant clinical challenge for sleep specialists. This CCS provides valuable guidance for defining, preventing, and addressing surgical failures in the treatment of OSA syndrome.

## Introduction

Obstructive sleep apnea (OSA) syndrome is a respiratory sleep disorder characterized by a reduction (hypopnea) or complete cessation (apnea) of airflow through the upper airways, in presence of breathing efforts, occurring during the night [1–4]. It is a common yet often undiagnosed condition, with an incidence rate ranging from 5 to 17% among middle-aged individuals and from 20 to 60% in those older than 65 years [5–8]. OSAS is becoming increasingly recognized due to its significant negative impact on daily life, including daytime sleepiness, neurocognitive issues, and psychological problems such as memory impairment, attention deficits, executive function disturbances, and depression [9–12].

The complexity of OSA, marked by its varied pathophysiology, phenotypes, and clinical presentations, has long presented a challenge to the medical community in determining the optimal treatment modality. Currently, continuous positive airway pressure (CPAP) therapy is considered the primary treatment option. However, despite its high success rate, CPAP compliance remains poor. According to the literature, adherence rates to CPAP therapy typically range from 30 to 60%, with continuous usage not exceeding 60% among compliant patients [12–18].

Upper airway (UA) surgery is often recommended for OSA patients who refuse or cannot tolerate CPAP therapy. The primary aim of surgery is to reduce apnea/hypopnea events by expanding, stabilizing, or removing obstructive tissue at various levels of the UA [19–21]. Over the years, numerous surgical interventions have been proposed, both at single and multi-level settings, including nasal surgery, velo-oro-pharyngeal surgery, tongue and base of tongue surgery, and maxillo-facial surgery. Despite advancements in techniques and technologies such as transoral robotic surgery, coblator, barbed sutures, surgery has not consistently proven effective, with reported success rates varying from 50 to 80% across different literature studies [21–25].

The intricate pathophysiology of OSA, diverse sites and patterns of UA collapse, and the interplay between anatomical and non-anatomical factors in each patient may contribute to surgical failure [26]. Thus, given the existing knowledge gap regarding factors associated with surgical success in adult OSA patients and the management of cases where surgery fails, we have chosen this topic for the development of an expert consensus statement (ECS). The objective of this ECS is to identify areas of agreement among a development group comprising international experts in the field of OSA surgery, focusing on defining surgical failure, identifying predictive factors linked to surgical failure, and outlining management strategies for such cases.

## Methods

The clinical consensus statement (CCS) was developed following the modified Delphi protocol proposed by Rosenfeld et al. [27]. Given the nature of the study, specific approval from an internal review committee was not required. The focus of the CCS was to establish specific guidelines for the definition, prediction, and management of surgical failure in OSA treatment.

This CCS was developed through the following steps: (1) Panelists’ Selection and Purpose of the Consensus Statement, (2) Literature Review, (3) Clinical Statement Development and Modifications in the Delphi Survey, (4) Revision of the CCS in an iterative fashion based on survey results, and (5) Data aggregation for analysis and presentation. The pertinent details of these steps are briefly described.

### The ECD development group selection and purpose of the consensus statement

The ECD development group comprised a chair (GI), an assistant chair (CV), and a methodologist (TM). Panelists were recruited voluntarily based on their clinical and research interests in surgical OSA treatment. The ECD development group consisted of 35 panelists (33 otolaryngologists, 2 sleep apnea surgeons) from Europe, North America, South America, and Asia. The ECS development group included representatives from the World Sleep Society, American Academy of Sleep Medicine, European Sleep Research Society, Asian Society of Sleep Medicine, and International Surgical Sleep Society. All members of the development group were experts in sleep apnea surgical treatment, actively involved in sleep medicine, and committed to participating in all verbal discussions (conducted via teleconference) and votes. No panelists reported any potential conflicts of interest.

### Literature review and determination of the scope of the consensus statement

A systematic literature review, following the Preferred Reporting Items for Systematic Reviews and Meta-Analyses (PRISMA) guidelines, was conducted between June 2023 and September 2023. Multiple databases (MEDLINE, EMBASE, Scopus, and Web of Science) were explored, using the following search terms: OSA surgical success rate, surgical results in OSA treatment, surgical failure in OSA treatment, factors related to surgical success in OSA treatment, factors related to surgical failure in OSA treatment, OSA surgical success improvement, diagnosis of OSA surgical failure, treatment in OSA surgical failure.

Three hundred and fifty-two articles were initially identified through the database search. After excluding 257 articles with low evidence based on recommendations from Rosenfeld et al.'s clinical consensus statement, the selection was narrowed down to randomized controlled trials, guidelines, and systematic reviews. Subsequently, out of the 73 articles left, 35 were removed after full-text examination, as they did not pertain to surgical success rates or failures in OSA treatment. The remaining 38 articles were compiled and distributed to all CCS authors for review over a period of two months.

**Fig. 1:**
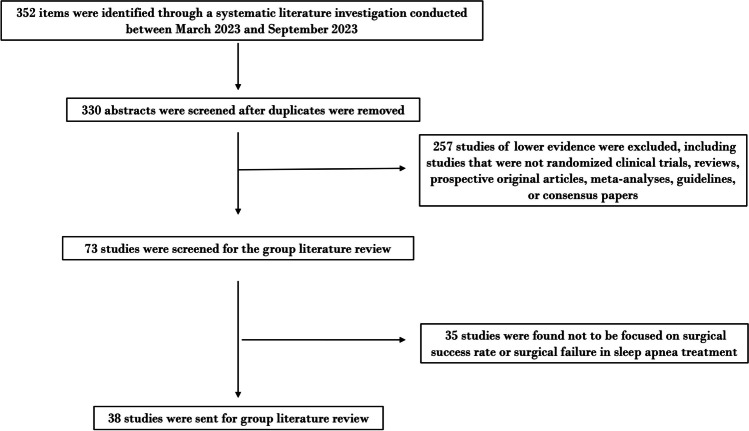
PRISMA-style flowchart of the article selection process

The article selection process is summarized in the PRISMA flowchart (Fig. 1), and the list of selected articles is included in Appendix.

This systematic review was developed to evaluate just the upper airway surgery and not included the Hypoglossal nerve stimulation therapy.

#### Clinical statement development and creation of the Delphi survey

The chair and assistant chair formulated the core clinical statements for the survey based on the objectives of the CCS and findings from the literature review. These statements were further refined and expanded by the methodologist. A total of 60 statements were compiled, incorporating insights from the literature review and the study group's evaluation of relevant clinical scenarios.

The initial draft of the survey was circulated among panelists, who were encouraged to propose modifications or suggest entirely new statements that they deemed pertinent to the CCS. Personal contacts from the chair or co-chair, along with group emails, were utilized to ensure comprehensive participation and representation of diverse viewpoints. No modifications or new statements were proposed initially.

Subsequently, a final 60-statement survey was developed and distributed to the authors using Google Forms (Google LLC, Mountain View, CA, USA). These 60 statements were categorized into sections addressing the definition of surgical success/failure, predictive factors of surgical failure, diagnostic workup, and treatment in cases of surgical failure.

Authors were instructed to anonymously complete the survey via the provided personalized single-use link. Each author rated their level of agreement with each statement using a 9-point Likert scale (ranging from 1 -strongly disagree- to 9 -strongly agree-), with the opportunity to anonymously express additional opinions after voting for each item.

The results for each statement were defined as follows:

- Strong consensus: Mean score of ≥ 8.00 with no outliers (defined as any rating 2 or more Likert points from the mean in either direction);
- Consensus: Mean score of ≥ 7.00 with no more than 1 outlier;
- Near consensus: Mean score of ≥ 6.50 with no more than 2 outliers;
- No consensus: All other statements.

Two iterations of the Delphi survey were conducted, with all group members fully participating in both rounds. Following the first round, 4/60 statements reached a strong consensus, 12/60 statements reached a consensus, 14/40 statements reached a near consensus, and 30/60 statements reached no consensus. Items with a mean score higher than 7 with no more than 1 outlier were dropped from the CCS and considered for the definitive consensus. Statements meeting the criteria for strong or consensus agreement were retained for the definitive consensus. The remaining 44 statements, showing near- or no consensus, were rephrased based on anonymous feedback comments from the authors to enhance inclusivity and clarity. These revised statements were then reintroduced in the second Delphi round.

During the second survey round, 2/44 items reached a strong consensus, 18/44 items obtained consensus. At the second round 24/44 items showed an average value < 7 or more of two outliers and were finally considered as no-consensus statements. Therefore, statements that attained strong consensus or consensus agreement were identified. However, those failing to meet the predefined consensus criteria were designated as no consensus statements. As no items reached near consensus in the second round, no further iterations of the Delphi survey were deemed necessary.

The final version of the ECSs was structured into distinct areas: the definition of surgical success/failure, predictive factors of surgical failure, diagnostic workup, and treatment in cases of surgical failure. The manuscript underwent collaborative drafting and final review by all development group members.

## Results

All panelists actively participated in the three Delphi rounds. Among the initial 60 statements, 6 achieved a strong consensus, 31 reached consensus, while 23 failed to garner any consensus.

The finalized version of all 60 statements, along with their mean and median scores, and the number of outlier scores, are presented in Tables 1, 2, 3, and 4 for items achieving strong consensus and consensus, subdivided across the 5 survey sections, and Table 5 for items without consensus. The statements are reported in the latest version as proposed in the CCS.

**Table 1:** Statements and results from the Delphi process for items reaching consensus or strong consensus: definition of surgical success/failure

Item No	Final Statement Version	Mean	Outlier	Delphi Round
3	The often-quoted Sher's criteria based only on numeric values of PSG test should be abandoned as they are both insufficient and out of date	8,04	0	I
4	The SLEEP-GOAL proposed by Pang and Rotemberg is a good, holistic and more comprehensive method to evaluate surgical success but difficult to use everything in daily clinical practice	7,57	0	II
5	New and precise criteria for defining indices of surgical success/failure in patients with OSA, taking into account polysomnographic parameters, reduction in symptoms and improvement in quality of life, should be defined	8,69	0	I
7	Improving patients' tolerability to CPAP reducing its positive pressure values, which would be achieved by reducing the severity of disease post-surgery, could be considered a realistic indicator of surgical success	7,07	1	II
10	Reported snoring after surgery is not indicative of surgical failure	7,14	0	II

**Table 2:** Statements and results from the Delphi process for items reaching consensus or strong consensus: predictive factors of surgical failure

11	Surgical failure in OSA can be attributed to multiple factors, including the incorrect patient selection, surgical planning, or postoperative care	8,65	0	I
12	Severity of the disease might be associated with a higher risk of surgical failure	7,35	1	I
14	Patient’s higher BMI increase the risk of a surgical failure	8,32	0	II
15	Advanced age is associated with an increased risk of surgical failure	7,1	1	II
16	Multilevel airways sites of obstructions increase the risk of a surgical failure, regardless of the type of surgery performed	7,4	0	II
17	The choice of surgical approach based on the patient's anatomy reduce the surgical failures	7,62	1	I
18	The preoperative DISE evaluation, identifying sites and pattern of collapse/obstruction, could reduce a possible surgical failure	7,54	1	II
20	The surgeon's experience and skills play a crucial role in reducing surgical failures	8,12	0	I
21	The choice of a surgical treatment agreed upon by a multidisciplinary team could reduce the risk of a surgical failure	7,19	1	I
23	Inadequate adherence of patients to the postoperative instructions can increase the risk of surgical failure	7,04	1	I

**Table 3:** Statements and results from the Delphi process for items reaching consensus or strong consensus: predictive factors of surgical failure: diagnostic workout

Item No	Final Statement Version	Mean	Outlier	Delphi Round
24	The Home Sleep Apnea Test (HSAT) study is an adequate test for the post-surgical evaluation of OSA	7,6	1	II
25	In case of persistence of symptoms post-surgery other reasons for excessive daytime sleepiness apart from sleep related disorders should be investigated	8,50	0	II
26	The first PSG study should be scheduled minimum 3-months after soft tissue surgery and 12-months after skeletal surgery	7,58	0	I
27	The postoperative PSG study should be performed with the same device used for the preoperative evaluation and in the same environmental situations	7,50	0	I
29	It is essential to investigate the post-operative ESS score in each surgical case	7,14	1	II
30	type I or II sleep study are suggested in case of post-surgery residual snoring and OSAS symptoms (e.g. ESS > 10) but normal AHI < 5 (HSAT)	7,1	1	II
32	DISE should be considered as valid test to identify sites of collapse and obstruction and should be considered in the diagnostic workout of patients with PSG-diagnosed surgical failure	7,50	1	I
33	Postoperative variation in number of apnea compared with the number of hypopneas instead of the comprehensive AHI value should be evaluated in any case of suspected surgical failure	7,23	0	I
34	The PALM evaluation should be considered in patients with surgical failure	7,23	1	I

**Table 4:** Statements and results from the Delphi process for items reaching consensus or strong consensus: predictive factors of surgical failure: treatment in surgical failure cases

Item No	Final statement version	Mean	Outlier	Delphi Round
37	In case of surgical failure, a multidisciplinary (pneumologist – dentistry – neurologist etc.) discussion is recommend to choose the best treatment	7,18	1	II
38	Patients with post surgery improvement of clinical symptoms, SpO2 outcomes and quality of life, even in the presence mild OSA (AHI between 5 and 15) could be managed with a clinical observation	7,88	0	I
39	Nasal surgery (septoplasty + turbinoplasty) is an effective treatment in symptomatic patients, surgically treated with a single level pharyngoplasty and postoperative residual mild OSA, with nasal obstruction and without a suspect of tongue base collapse	7,50	1	II
44	Mandibular advancement device might be an effective treatment in case of a single level pharyngoplasty surgery failure and postoperative base of tongue collapse	7,75	1	II
46	Positional devices are effective treatments for surgically treated patients with a residual post-surgical OSA and PSG features of POSA	7,23	1	I
47	Radiofrequencies on the soft palate are reasonable and effective procedures in patient with postoperative AHI < 5 and residual snoring	7,54	1	II
51	Base of tongue surgery is a reasonable and effective treatment in patients surgically treated with a single level pharyngoplasty and base of tongue collapse with lymphoid tissue hypertrophy	7,86	0	II
54	Maxillomandibular advancement (MMA) surgery or hypoglossal nerve stimulation are possible surgical treatment in a retrognatic patient with residual OSA, previously surgically treated with a single level pharyngoplasty surgery	7,89	0	II
55	Maxillomandibular advancement (MMA) surgery or hypoglossal nerve stimulation surgery are reasonable and effective in patients surgically treated with a multilevel surgery and residual OSA with multilevel obstructions	7,64	1	II
58	Epiglottis surgery (epiglottoplasty or epiglottopexy) is a reasonable and effective treatment in patients surgically treated with a single level pharyngoplasty and postoperative epiglottis collapse	7,64	1	II
59	Hypoglossal nerve stimulation is a safe and effective procedure in residual OSA patients eligible for this surgical treatment	7,69	0	I
60	Medical therapy is safe and effective to treat residual symptoms (ESS > 10) in and residual OSA in patients with identified non anatomical pathophysiological traits	7,4	1	II

**Table 5:** Statements and results from the Delphi process for items not reaching consensus

Item No	Final statement version	Mean	Outlier	Delphi Round
Definition Of Surgical Success/Failure				
1	A Postoperative Home Sleep Test showing AHI value greater than 5, with associated daytime and/or night-time OSA symptomatology, is indicative of surgical failure	4,34	3	II
2	The frequently cited Sher’s criteria (50% improvement in AHI < 20 and AHI < 20) are the benchmark to define surgical success	3,92	4	II
6	Persistence of sleep apnea symptoms (daytime sleepiness, morning headache, memory impairment) equal to the preoperative evaluation are indicative of surgical failure	7,19	3	II
8	The PSG study is needed post surgery only if the patient has persistent symptoms of OSA	4,8	4	II
9	Patient with a postoperative long-term complications (e.g. dysphagia, open rhinolalia, throat pain) without symptoms and with AHI value < 5, is considering a surgical failure	5,29	3	II
Predictive Factors Of Surgical Failure				
13	Patient’s comorbidities (cardiovascular diseases or diabetes) are associated with a higher risk of surgical failure	6	2	II
19	The presence of sleep position-dependent OSA can positively influence the surgical outcome	5,7	2	II
22	Intraoperative and postoperative complications, such as excessive bleeding, increase the risk of a surgical failure	7,3	3	II
Diagnostic Workout				
28	In case of post-operative HSAT study (not earlier than 3 months post-op) confirming similar preoperative data (surgical failure) a repetition of a second postoperative HSAT should be considered before confirming the diagnosis of surgery failure	6,2	3	II
31	In case of residual OSA post-surgery, the postoperative work-out should include logopedic evaluation for myofunctional therapy	6,3	2	II
35	Head and neck MRI should be performed in case of surgical failure	3,6	4	II
36	In case of surgical failure, further tests are not necessary and CPAP treatment should be directly recommended	3,1	3	II
Treatment In Surgical Failure Cases				
40	Nasal surgery (septoplasty + turbinoplasty) is an effective treatment in symptomatic patients, surgically treated with a single level pharyngoplasty and postoperative residual moderate-severe OSA, with nasal obstruction and without a suspect of tongue base collapse	5,9	4	II
41	In case of enlarged lower turbinates, in a patients with and allergic rhinitis, surgically treated with a single level pharyngoplasty and postoperative residual mild OSA, steroids are an effective treatment	6,3	3	II
42	CPAP therapy is the only valid therapeutic option in case of surgical failure of a single level pharyngoplasty	2,8	5	II
43	CPAP therapy is the only valid therapeutic option in case of surgical failure of a multilevel surgery	4,1	2	II
45	Mandibular Advancement Device might be an effective treatment in patients surgically treated with a multilevel surgery and postoperative multilevel obstruction	5,4	4	II
48	Revision pharyngoplasty with a lateral pharyngoplasty (e.g., barbed relocation pharyngoplasty or expansion sphincter pharyngoplasty) is a reasonable and achievable procedure in patients previously surgically treated with Uvulopalatopharyngoplasty – UPPP, with residual OSA and lateral velo-pharyngeal collapse	6,5	0	II
49	A revision surgery with a UPPP is safe and effective in patients with residual OSA previously surgically treated with a barbed relocation pharyngoplasty	3,3	3	II
50	Myofunctional therapy is an effective treatment in patients surgically treated with a single level pharyngoplasty with lower muscular tone and mild-moderate residual OSA	7,1	3	II
52	Base of tongue surgery is a safe and effective treatment in retrognathic patients surgically treated with a single level pharyngoplasty surgery with postoperative base of togue collapse	5,6	3	II
53	Revision multilevel surgery (velo-pharyngeal + base of tongue surgery) is safe and effective in patients surgically treated with multilevel surgery showing postoperative multilevel collapse	5,3	3	II
56	Midline glossectomy is a reasonable and effective treatment in patients surgically treated with a single level pharyngoplasty surgery and postoperative oral tongue collapse	5,14	3	II
57	Midline glossectomy is a reasonable and effective treatment for patients with persistent OSA after multilevel surgery and oral tongue collapse	4,8	2	II

Regarding the ‘Definition of Surgical Success/Failure after OSAS surgery’ (Table 1), 5 out of 10 statements reached consensus, while the remaining 5 did not (Table 5). There was extensive discussion within the development group regarding the necessity for an appropriate definition of surgical failure after OSA surgery. Emphasis was placed on the importance of establishing new and precise criteria to define surgical success rates or failures after OSA surgery.

Regarding the ‘Predictive Factors of Surgical Failure in OSAS surgery’ (Table 2), 10 out of 13 statements reached consensus, with the remaining 3 failing to do so (Table 5). The CCS group agreed that surgical failure in OSA could stem from various factors, including incorrect patient selection and surgical planning, inadequate postoperative care, disease severity, higher BMI, advanced age, and involvement of multiple airway sites. Additionally, consensus was reached on the potential benefits of preoperative DISE, multidisciplinary preoperative evaluation, and surgeon experience and skills in increasing surgical success rates.

Regarding the ‘Diagnostic Workup in OSAS surgery’ (Table 3), 9 out of 13 statements reached consensus, while the remaining 4 did not (Table 5). The CCS group agreed that the Home Sleep Apnea Test (HSAT) is suitable for post-surgical evaluation of OSA and recommended scheduling the first PSG study at least 3 months after surgery.

Regarding the ‘Treatment in Surgical Failure Cases’ (Table 4), 12 out of 24 statements reached consensus, whereas the remaining 12 did not. The development group underscored the importance of a multidisciplinary discussion involving pneumologists, dentists, neurologists, etc., to select the optimal treatment in cases of surgical failure. They emphasized that CPAP is not the sole treatment option in such instances, advocating for consideration of alternative therapeutic solutions based on the patient's clinical and pathophysiological characteristics.

## Discussion

### Surgery and selection criteria

Various surgical procedures have been developed over the years to increase the upper airway airspace in OSA patients [26–67]. However, no surgical treatment is 100% effective, and many have shown limited clinical application due to lower effectiveness in reducing hypopnea/apnea events or a higher incidence of related comorbidities. Surgical interventions should be carefully selected based on the patient’s anatomy, clinical characteristics, sites of obstruction, type of collapse, clinical severity of OSA, and the potential complications of each surgical technique [33–41, 58–61].

Drug-induced sleep endoscopy is now a validated tool for identifying the specific sites of obstruction and the type of collapse. Using this tool, it is possible to determine which patients are candidates for oropharyngeal, base of tongue, hypopharyngeal, and epiglottic surgery. Each of these procedures may be performed alone or as part of a multilevel approach, depending on the identified sites of collapse [45].

Below, we outline the most commonly used and widespread surgical techniques, along with their respective indications, which should be considered as a starting point for this consensus statement on surgical failure.

#### Velo-oropharyngeal surgery

This is used in cases of isolated velo-oropharyngeal obstruction/collapse or in combination with base of tongue/epiglottis surgery in patients with multilevel obstruction sites.

##### Ablative surgery

Uvulopalatopharyngoplasty (UPPP) was the first oropharyngeal surgery described for treating snoring and OSA and remains a surgical milestone. The general principle of this ablative procedure is to enlarge the retropalatal dimension. However, the overall success rate for mild to severe OSA is reported to be between 35 and 50%, according to various studies. UPPP is generally a painful procedure, associated with complications such as nasopharyngeal stenosis and incompetence. Due to these reasons, conservative modifications and non-ablative techniques have been developed in recent years, leading to a decline in its use among sleep surgeons [16–25, 45–58].

##### Reconstruction of the lateral pharyngeal wall (Pharyngoplasty)

This procedure is designed to prevent airway collapse by stabilizing the lateral pharyngeal wall. Techniques such as lateral pharyngoplasty and expansion sphincter pharyngoplasty, introduced in the 2010s, have shown a good surgical success rate ranging between 57 and 86%. These techniques also reduce various comorbidities associated with ablative procedures [18–35].

##### Suspension techniques

In recent years, palatal surgery has increasingly focused on techniques that suspend the palatopharyngeus muscle to the pterygomandibular raphe. These surgeries aim to enlarge the velopharyngeal airspace and stabilize the lateral walls to prevent collapse. Techniques such as the Barbed Roman blinds technique, barbed anterior pharyngoplasty, barbed reposition pharyngoplasty, suspension palatoplasty, and barbed suspension pharyngoplasty have been proposed. These have shown a surgical success rate between 50 and 80%, according to recent studies, with a much lower incidence of postoperative comorbidities [35–45].

#### Base of tongue and epiglottis surgery

It is rare to observe an isolated collapse of the tongue base, so this treatment is usually combined with palate and/or epiglottis surgery. Transoral robotic surgery (TORS) is currently considered one of the most effective procedures for treating patients with base of tongue obstruction. This technique allows hypopharyngeal enlargement through tongue base reduction. In cases of secondary epiglottis collapse, a resection of the free edge of a floppy epiglottis could be performed by TORS. Recent meta-analyses have shown excellent outcomes, with significant improvement achieved in 70–85% of patients undergoing TORS in combination with various palatal surgeries [68].

Recently, some authors have reported the use of coblator technology to perform base of tongue and epiglottic resection, with results comparable to TORS in terms of AHI reduction and postoperative outcomes. A midline glossectomy using coblator technology is another surgical option when oral tongue collapse is evident, with 56% of patients showing significant postoperative improvement in AHI scores [25–35, 52].

Midline glossectomy via an open approach, along with other tongue/hypopharyngeal surgeries such as tongue suture suspension, genioglossus advancement, hyoid suspension, tongue base radiofrequency ablation, and CO2 laser lingual tonsillectomy, were not considered in this discussion. These procedures are less commonly used in clinical practice and have shown inconsistent results in the literature [52, 55–60].

Isolated epiglottic collapse can be treated with various techniques, such as epiglottis laser resection, stiffening operations, and epiglottopexy, all of which have demonstrated good functional and respiratory outcomes. These techniques have shown similar postoperative results in treating primary epiglottic collapse [52].

#### Skeletal surgery

Maxillomandibular advancement (MMA) is considered the most successful surgical procedure for OSA after tracheotomy. It offers airway expansion and associated soft tissue distraction at multiple levels, with positive long-term follow-up results. A recent systematic review and meta-analysis showed that MMA is highly effective in treating OSA, with the mean AHI decreasing from 63.9/h to 9.5/h (p < 0.001) and a pooled surgical success rate of 86.0%. However, due to the changes in facial physiognomy, it is typically considered for adults with retrognathia or craniofacial deformities [52, 58–64].

#### Neurostimulation

Hypoglossal nerve stimulation (HNS) is an implantable device that stimulates the hypoglossal nerve to activate upper airway dilator muscles (e.g., genioglossus), improving airway patency without waking the patient. It is indicated for moderate to severe OSA patients with multilevel obstruction, excluding those with retrovelar-circular collapse. HNS can significantly improve moderate to severe OSA in patients who are nonadherent to PAP therapy. Approximately 70%–75% of patients significantly reduce the severity of OSA using HNS [52, 65–67].

### Definition of surgical success/failure

The gold standard diagnostic test for OSA is overnight polysomnography (PSG), with diagnosis and severity typically assessed based on the apnea–hypopnea index (AHI), representing the number of apnea and hypopnea events per hour. The evaluation of effectiveness of each single or combined surgical interventions for OSA almost exclusively relies on reported changes in AHI post-operatively [28, 29]. While the development group agreed on the necessity of post-operative PSG to assess surgical success, they emphasized that PSG results should be carefully interpreted and not solely relied upon. The AHI is a measure susceptible to extreme variability due to one-night sleep study, the first night-effect, patient anxiety, the restriction of movements from the abundance of monitoring wires. Besides, recent evidence has shown that there is a consistent discordance between the levels of AHI used to denote outcomes/success of therapy and real-world clinical outcomes such as quality of life (QoL), patient perception of disease, and cardiovascular measures (e.g., blood pressure, oxygen saturation) [28–31].

Extensive discussion within the CCS group underscored the need for a more comprehensive definition of surgical failure beyond post-operative AHI values. The group expressed reservations about using exclusively post-operative AHI values, declaring they should not be the only parameters defining surgical success as the often-quoted and reported Sher's criteria, which are based solely on PSG numeric values (50% reduction in AHI and an AHI below 20): they are insufficient and do not adequately consider clinical aspects. Instead, the group advocated for the development of new, holistic and comprehensive criteria encompassing AHI reduction, symptom improvement, physical findings, and QoL enhancement [25, 29, 33].

In many areas of medicine, patient-reported outcome measures and quality of life (QoL) assessments are increasingly recognized as essential for evaluating the effectiveness of therapies for various diseases. To redefine what constitutes successful treatment in obstructive sleep apnea (OSA), several authors have suggested that subjective measurements—such as QoL scores, sleepiness assessments, performance testing, physical findings, and patient perceptions of their condition—should be considered. These clinical findings are argued to better reflect the impact and manifestations of OSA on patients than the simple Apnea–Hypopnea Index (AHI) [1–15, 30–35].

In a clinical study, Pang and Rotemberg proposed a new score to define surgical success in OSA surgery, which they called the SLEEP GOAL [33]. The SLEEP GOAL is an acronym related to the end-organ effects in OSA patients. It measures the cardiovascular and neurocognitive effects of the OSA disease process as well as the overall disease burden. To demonstrate the efficacy of the SLEEP GOAL in evaluating the success rate of surgically treated sleep apnea patients, they analyzed 302 patients treated for OSA across nine tertiary clinical centers in seven countries. The overall success rate, based on the Sher criteria, was 66.2%. The success rate based on the SLEEP GOAL was 69.8%. If evaluated solely on the Sher criteria, 63 patients with significant blood pressure reduction, 29 patients with BMI reduction, and 66 patients with a clinically significant decrease in the duration of oxygen saturation < 90% would have been misclassified as "surgical failures" [33].

The CCS group found the SLEEP GOAL score to be a holistic and more comprehensive approach, worthy of consideration. However, despite its thorough evaluation of surgical success, there is a common perception that this score is still rarely applied in clinical practice due to the numerous parameters required to calculate it.

Upper Airway Surgery (UAS) has shown potential in reducing positive CPAP pressure values and improving adherence to it [34–36]. Ayers et al.'s [37] systematic review and meta-analysis demonstrated a significant reduction in CPAP levels after UAS in the majority of studies analyzed. The CCS group acknowledged that enhancing patient tolerability and adherence to CPAP, along with reducing positive pressure values, could serve as realistic indicators of surgical success.

In summary, the CCS group emphasized the need for a broader definition of surgical success in OSA treatment, considering not only AHI reduction but also improvements in symptoms, physical findings, and QoL. The SLEEP GOAL score and the impact of UAS on CPAP levels and adherence were highlighted as promising avenues for evaluating surgical success.

### Predictive factors of surgical failure

The CCS group strongly emphasized that surgical failure in OSA can be attributed to various factors, including incorrect patient selection, surgical planning, or postoperative care. Additionally, after deliberation, the group identified disease severity, higher BMI, advanced age, and multilevel airway obstructions as closely associated with potential surgical failure [23–35, 38–41]. A correlation between these patient’s characteristic and surgical outcome was recently evaluated by Kim et al. [42] using a machine learning model; using a logistic regression model the age, lowest O2 level, AHI value, tonsil size and multilevel sites of obstruction were considered as the major contributors to surgical outcomes. Higher BMI was also inversely related with the surgical success rate in the random forest and gradient boosting models.

The CCS panelists stressed the importance of tailoring the surgical approach to each patient's upper airway anatomy. Patients with specific anatomical characteristics, such as pronounced macroglossia, high Mallampati score of IV, Friedman oral tongue score of III-IV, marked tongue/tongue-base hypertrophy or retrognathic patients, may not benefit from isolated velo-pharyngeal or tongue surgeries due to potential obstructions in other UA sites, beyond the velo pharyngeal region [20–25, 42–44]. The same, patients with a high, upright and muscular tongue may not benefit from a basic tongue resection (by TORS, COBLATOR etc.). Besides, there was a strong consensus that a preoperative Drug Induced Sleep Endoscopy (DISE) could mitigate a possible surgical failure by identifying the real UA obstruction sites collapse patterns in each OSA patient candidate to surgery. These aspects have been recently confirmed in a randomized study of Iannella et al. [45].

The ECS group suggest that sll these patient’s characteristics should be carefully considered by a watchful sleep surgeon before performing any type of surgery [42–45].

Considering these factors, the CCS group recommended a multidisciplinary team approach involving pneumologists, ENT specialists, and odontostomatologists to determine the optimal treatment for each patient and minimize surgical failure. The collaborative effort allows for a comprehensive understanding of OSA phenotypes, facilitating the selection of appropriate surgical interventions [46, 47].

Although OSA patients commonly suffer from systemic comorbidities such as cardiovascular diseases or diabetes, the CCS group was not able to find any correlation between these and surgical failure. Besides, no studies published in literature correlated these preoperative patients’ comorbidities with results of the OSA surgery. Therefore, according to the experts clinical experience, patients with systemic conditions should not be excluded from OSA surgery based solely on their comorbidities. Similarly, intraoperative and postoperative complications, such as bleeding, long term dysphagia or suture extrusion, were not found to increase the risk of surgical failure in relevant clinical studies48- [50]. In a clinical study of Gulutta et al. [51] regarding barbed pharyngoplasties no correlation between suture extrusion or exposition emerged. The same Moffa et al., [49] in a systemic review on complications and side effects after barbed pharyngoplasty, did not report any correlation between these and the lack of gain on the nocturnal respiratory parameters of the OSA.

Regarding the patients suffering of positional dependent OSA (POSA), there was extensive discussion if they could better respond to surgery. While some authors suggested better surgical outcomes for velo-pharyngeal surgeries in POSA patients, the CCS group did not reach a consensus [52–54]. Data from a clinical retrospective study of Cammaroto et al. [55] indicated that non-positional OSA patients might benefit more from barbed repositioning pharyngoplasty (BRP) procedures compared to positional patients, highlighting the complexity of treatment response across different OSA subtypes.

### Diagnostic workup

The CCS group unanimously agreed that the Home Sleep Apnea Test (HSAT) is an appropriate tool for post-surgical evaluation of OSA. They emphasized that HSAT should be used as part of a comprehensive sleep evaluation rather than as a stand-alone test. The panel recommended scheduling the HSAT at least 3 months after soft tissue surgery to allow sufficient time for healing and to obtain accurate results. Conversely, the ECS suggested that for skeletal surgeries, such as maxillomandibular advancement (MMA), the best results are typically observed up to 12 months post-surgery due to ongoing musculoskeletal remodeling.

They also recommended assessing postoperative variations in the ratio of apnea to hypopnea events rather than relying solely on the AHI value, particularly in cases of suspected surgical failure, to accurately gauge surgical success.

In alignment with AASM guidelines [52, 56], the CCS group strongly supported clinical follow-up with a polysomnography study (PSG Type I or II) if OSA symptoms persist despite compliance with appropriate therapy. Specifically, in the context of surgical patients exhibiting residual symptoms (Sleepiness, morning headache, concentration deficit, etc.) alongside normal AHI values postoperatively at the HSAT test, the group deemed a Type I or II sleep study necessary to rule out neurological diseases or other sleep-related disorders.

Drug-induced sleep endoscopy (DISE) emerged as a valuable postoperative diagnostic tool for patients with suspected surgical failure, allowing identification of untreated UA obstruction/collapse sites, or treated areas where surgery has not been effective. The group endorsed DISE as a safe and effective procedure with minimal risks, therefore it should be considered in the postoperative diagnostic workup of patients with PSG-diagnosed surgical failure [45, 55, 57].

The pathophysiology of OSA is multifactorial, resulting not only from impaired upper airway collapsibility/obstruction (anatomical factors) but also from various non-anatomical factors. These factors, when combined, contribute to different OSA physiological traits (PT) [1–15, 27–33]. These traits include: 1) upper airway (UA) obstruction and collapsibility, which can be measured using the critical occlusion pressure (Pcrit), defined as the endo-pharyngeal pressure associated with UA collapse; 2) a poor ability of the upper airway muscles to respond to respiratory challenges by stiffening or dilating the airway; 3) a low respiratory arousal threshold, causing an individual to wake from sleep with only a small increase in respiratory drive; and 4) a hypersensitive ventilatory control system, often referred to as a system with a high loop gain [35–37].

The PALM scale (Pcrit, arousal threshold, loop gain, and muscle responsiveness) has been developed to better stratify OSA patients based on the prevalence of these anatomical and non-anatomical factors. This approach helps avoid ineffective treatments in patients where non-anatomical traits predominate. Indeed, when non-anatomical features are present, even with a reduction in collapse pressure, the patient may not experience an improvement in the number of apneas/hypopneas [30–38, 58].

In summary, when moderate-to-severe anatomical problems with higher collapsibility and elevated Pcrit are present (PALM 1 and 2a), CPAP treatment or other interventions that modify the upper airway or its collapsibility (such as surgery, mandibular advancement devices, positional therapy, or lifestyle changes) are indicated. As non-anatomical factors become more significant than upper airway anatomy, one or more interventions targeting each specific physiological impairment should be considered. These might include treatments to improve upper airway muscle function, such as upper airway muscle stimulation, medications to reduce loop gain with acetazolamide, or increasing the arousal threshold with hypnotics [26–28, 58].

Unfortunately, measuring the PALM variables remains challenging in a clinical setting, as it requires full polysomnography and subsequent processing of large amounts of data. However, some surrogates to evaluate Pcrit and the PALM score have been proposed by clinical and experimental studies. According to data published by Eckert et al. [58] and Bosi et al. [26], it may be possible to use AHI severity and the therapeutic pressure level of CPAP to estimate the value of Pcrit, allowing for a more accurate subclassification of OSA patients.

In addition to the anatomical collapsibility classification, a qualitative assessment of other physiological traits (LG and AT) can be achieved using a similar strategy. Table 6, derived from the study by Bosi et al. [26], illustrates how to subclassify PALM scores according to PSG and CPAP data, highlighting the key parameters and PSG patterns for LG and AT qualitative phenotyping.

**Table 6:** PALM stratification according to AHI and CPAP values as described by Eckert et al. and Bosi et al. [26]

Collapsibility	AHI	CPAP Value	PALM Classification
less than − 2.5 cmH2O	< 40	≤ 8 cmH2O	PALM 3
between − 2.5 and + 2.5 cmH2O	< 40	> 8 cmH2O	PALM 2
more than − 2.5 cmH2O	> 40	> 8 cmH2O	PALM 2 or 1
Ventilatory Instability		Arousal Threshold (AT)	
(1) Coexistence of OSA and CSR (2) High proportion of central/mixed respiratory events (3) NREM dominant pattern (CAP dominant OSA)		Low AT (1) At least 2/3 variables (AHI 58.3%, SaO2 Nadir > 82.5%) High AT (2) Long event duration and severe desaturations (e.g., OHS)	

By using the AHI (cut-off 40 events/h), in parallel with the effective pressure value (CPAP ≤ 8 cm), it is possible to characterize the collapsibility of relatively high percentage of OSA patients. *AHI* apnea hypopnea index, *CPAP* continuous positive airway pressure, *OSA* obstructive sleep apnea, *PALM* stands for critical pressure, A for arousal threshold, L for loop gain, and M for muscle recovery, *AT* arousal threshold, *CSR* Cheyne–stokes respiration, *CAP* cyclic alternating pattern, *OHS* obesity hypoventilation syndrome

In light of this evidence, the CCS group emphasized the importance of evaluating the PALM scale to stratify OSA physiological traits. The CCS strongly promotes the use of the PALM score, particularly in patients who have experienced surgical failure. This approach aims to prevent further ineffective treatments in patients with a predominance of non-anatomical traits, ensuring an appropriate management strategy for this subgroup of patients [24–26, 50–60].

The CCS group did not find evidence of utility in neither logopedic evaluation for myofunctional therapy nor head and neck MRI in the postoperative diagnostic workup for residual OSA. These modalities were not deemed beneficial in addressing surgical failure according to available evidence and expert consensus.

In summary, the CCS group emphasized the importance of comprehensive post-surgical diagnostic evaluation, incorporating tools such as HSAT, PSG studies, DISE, and consideration of anatomical and non-anatomical factors using the PALM scale to guide treatment decisions and optimize outcomes for patients with suspected surgical failure.

### Treatment in surgical failure cases

Treating OSA after surgical failure poses a significant challenge for sleep surgeons. The CCS group strongly recommends, in cases of surgical failure, initiating a multidisciplinary team discussion involving pneumologists, dentists, otolaryngologists, neurologists, etc., to determine the best treatment based on the patient's clinical and instrumental phenotyping.

Among treatment options in surgically treated patients with residual OSA or complete surgical failure, different non-surgical and surgical therapies are available: ventilatory therapy, oral appliances, second look surgery and positional therapy are certainly the most viable solutions [24, 25, 48, 59, 60].

### Non-surgical treatment

#### Wait and see

It is not typically considered an actual option after a surgical failure. The main criterium which leads to this approach lies in the patient’s choice. In this case the patients must be fully informed about its OSA pathology, clinical consequences of an untreated condition and the possibility of other therapeutic solutions [48, 57]. According to the CCS group the "wait and see" approach may be suitable in selected cases where patients experience improvements in symptoms, SpO2 outcomes, and quality of life post-surgery, even with mild to moderate OSA (mainly for AHI values between 5 and 15).

#### CPAP therapy

The CCS panel advises against solely considering CPAP therapy in cases of single-level or multilevel surgical failure. However, the reduced nasal resistance and increased pharynx stability achieved through UA surgery may allow for lower CPAP pressure settings, potentially enhancing device acceptance and compliance [25, 52, 61].

#### Mandibular advancement devices (MAD)

MAD is one of the most widely used treatments for OSA syndrome. When appropriately customized and titrated, MADs can stabilize the upper airways in retro-palatal and retro-lingual areas [48]. While literature evidence supporting MAD use post-surgery is limited, it may be effective after some surgical failures [62, 63]. The CCS group recommends the use of MAD as an effective treatment in cases of single-level pharyngoplasty surgery failure with identified postoperative base of tongue collapse. However, following a long discussion, the development group advises against MAD usage as a first-line treatment in patients with persistent OSA who underwent multilevel surgery (pharyngoplasty + oral or base tongue surgery).

#### Positional therapy

The utility of Positional devices for patients with a residual post-surgical OSA and PSG features of POSA was confirmed by the CCG group after a long discussion. CCS group recommended to have a proper time window of observation in the lateral position and to classify with APOC scale the patients in order to identify possible responders to therapy [55, 64]. Last but not least, there is an increasing amount of evidence about the use of 30° Fowler’s position in managing positional OSA in a more comprehensive way.

#### Myofunctional therapy

Currently, there is limited data reported in literature on the effectiveness of post-operative Myofunctional therapy in OSA relapse [65, 66]. The CCS group was not able to identify it as an effective treatment in patients with lower muscular tone and mild to moderate residual OSA post-surgery, highlighting the need for further research studies in this area.

#### Drugs

Different drugs are under evaluation for the targeted treatment of the non-anatomical traits of OSA [57]. The CCS group of study agreed with the use of a Medical therapy to treat residual symptoms (ESS > 10) or in residual OSA in patients with identified non anatomical pathophysiological traits. This is a promising research field of interest, however it’s still requiring further studies to gain a sound place in clinical practice.

#### Revision-surgical treatment

The choice of surgical options to address SDB recurrence requires careful global reassessment including physical exam, biometrics, awake endoscopy and in most of the cases a DISE examination [45, 46]. The surgeon must identify the best procedure and the patient is requested to accept the suggested one.

Many surgeries may be considered as rescue procedures. Vicini and Cammaroto [48] reported the use of BRP as revision procedure in failed UPPP or ESP procedures. Despite these evidences and after an extensive discussion the CCS group disagreed in considering the revision lateral pharyngoplasties as a reasonable and achievable procedure in patients previously surgically treated with UPPP, with residual OSA and lateral velo-pharyngeal collapse. Differently, Base of tongue surgery has been considered as a reasonable and effective treatment in patients surgically treated with a single level pharyngoplasty and base of tongue collapse with lymphoid tissue hypertrophy. CCS group agreed that maxillomandibular advancement (MMA) surgery or hypoglossal nerve stimulation are possible surgical treatment in a retrognathic patient with residual OSA, previously surgically treated with a single level or multilevel pharyngoplasty surgery [52, 67].

#### Weight loss

Weight loss, diet/exercise, bariatric surgery and GLP-1 medications should also be considered in the overweight/obese patient before considering other treatments [1, 15, 25–29]. Overweight/obese patients are usually not initial candidates for surgery and therefore these treatments were not considered in the statements developed by the expert group.

#### Study strengths

This clinical consensus statement has been developed by experts with extensive knowledge and experience in sleep apnea surgery, lending significant credibility and authority to the recommendations. The CCS addresses a critical topic—management of surgical failure—where formal guidelines are currently lacking, thereby helping to fill important gaps in knowledge and clinical practice. Moreover, the CCS focuses on practical and real-world issues related to surgical failure, making it highly relevant for day-to-day clinical decision-making.

### Study limitations

The recommendations may reflect the opinions and experiences of the experts involved, which might not always be generalizable or applicable to all clinical settings. While valuable, consensus statements do not replace formal clinical guidelines, which are based on a more comprehensive analysis of the evidence.

## Conclusion

Surgical failures are a reality in the clinical management of OSA surgery, posing significant challenges for sleep specialists. Effectively treating these patients requires a deep understanding of OSA pathology and its treatment options. In this study, an expert group developed a consensus statement on OSA surgical failure, based on available evidence and expert opinion, using a Delphi method protocol. This consensus emphasizes the multifaceted nature of surgical failure in obstructive sleep apnea, underscoring the critical importance of patient selection and providing valuable insights into defining, preventing, and treating surgical failures in OSA syndrome.

This clinical consensus statement serves as a practical guide for all sleep surgeons, particularly those who are younger or less experienced. It offers practical advice on how to approach patients who have not achieved successful surgical outcomes. Through this CCS, we address controversial aspects found in both the literature and clinical practice regarding the diagnosis and management of these patients. The knowledge discussed in this CCS can help sleep doctors identify characteristics of patients at high risk for surgical failure. Understanding and proactively addressing predictors of surgical failure are essential for optimizing patient outcomes. Finally, the statement provides tailored suggestions on how to manage each patient who has experienced surgical failure, according to the specific type of surgery they have undergone.

## Data Availability

The data that support the findings of this study are available on request from the corresponding author.
